# Preparation of Laponite Bioceramics for Potential Bone Tissue Engineering Applications

**DOI:** 10.1371/journal.pone.0099585

**Published:** 2014-06-23

**Authors:** Chuanshun Wang, Shige Wang, Kai Li, Yaping Ju, Jipeng Li, Yongxing Zhang, Jinhua Li, Xuanyong Liu, Xiangyang Shi, Qinghua Zhao

**Affiliations:** 1 Department of Orthopaedics, Shanghai First People's Hospital, School of Medicine, Shanghai Jiao Tong University, Shanghai, P. R. China; 2 State Key Laboratory for Modification of Chemical Fibers and Polymer Materials, College of Materials Science and Engineering, Donghua University, Shanghai, P. R. China; 3 State Key Laboratory of High Performance Ceramics and Superfine Microstructure, Shanghai Institute of Ceramics, Chinese Academy of Sciences, Shanghai, P. R. China; 4 College of Chemistry, Chemical Engineering and Biotechnology, Donghua University, Shanghai, P. R. China; Texas A&M University Baylor College of Dentistry, United States of America

## Abstract

We report a facile approach to preparing laponite (LAP) bioceramics via sintering LAP powder compacts for bone tissue engineering applications. The sintering behavior and mechanical properties of LAP compacts under different temperatures, heating rates, and soaking times were investigated. We show that LAP bioceramic with a smooth and porous surface can be formed at 800°C with a heating rate of 5°C/h for 6 h under air. The formed LAP bioceramic was systematically characterized via different methods. Our results reveal that the LAP bioceramic possesses an excellent surface hydrophilicity and serum absorption capacity, and good cytocompatibility and hemocompatibility as demonstrated by resazurin reduction assay of rat mesenchymal stem cells (rMSCs) and hemolytic assay of pig red blood cells, respectively. The potential bone tissue engineering applicability of LAP bioceramic was explored by studying the surface mineralization behavior via soaking in simulated body fluid (SBF), as well as the surface cellular response of rMSCs. Our results suggest that LAP bioceramic is able to induce hydroxyapatite deposition on its surface when soaked in SBF and rMSCs can proliferate well on the LAP bioceramic surface. Most strikingly, alkaline phosphatase activity together with alizarin red staining results reveal that the produced LAP bioceramic is able to induce osteoblast differentiation of rMSCs in growth medium without any inducing factors. Finally, *in vivo* animal implantation, acute systemic toxicity test and hematoxylin and eosin (H&E)-staining data demonstrate that the prepared LAP bioceramic displays an excellent biosafety and is able to heal the bone defect. Findings from this study suggest that the developed LAP bioceramic holds a great promise for treating bone defects in bone tissue engineering.

## Introduction

Bone defects arising from trauma, tumor or bone-related diseases are causing more social issues due to the lack of ideal bone tissue substitutes [1]. Since a Dutch surgeon first used a piece of a dog's skull to repair a soldier's cranium in the 17^th^ century [2], repair of bone defect effectively using substitutes such as autografts and allografts has been of great importance. However, both of the traditional autografts and allografts are not the best candidates, since autografts may face to the donor shortage and donor site morbidity, whereas allografts may suffer the risk of disease transmission and immune response [3]. With the advances of tissue engineering and regenerative medicine [2], [4], a majority of damage to any tissue or organ is expected to be solved in clinic [5]. One of the most important issues in tissue engineering and regenerative medicine is to develop various artificial 3-dimensional scaffolds with appropriate physical and/or chemical properties that can closely mimic the natural extracellular matrix [6]. These scaffolds should not bring any immune or other adverse responses after implantation, should be porous in nature with high surface area to volume ratio to facilitate cell attachment, proliferation, and differentiation so that new tissue can be easily formed, and should be biodegradable so that they do not require any additional surgical procedures to be removed out of body [7].

Beyond polymer scaffolds, inorganic bioceramic materials have been received a great deal of attention for uses as implantation and/or fixation biomaterials [8]. Since the late 1960s, bioceramic has been used as alternatives to metals in order to increase the biocompatibility of the implants [9]. Bioceramic could be composed of several elements including alumina, zirconia, carbon, silica-contained compounds, and some other chemical ingredients [8]. Till now, bioceramic including bioactive glasses [10]–[12], sintered hydroxyapatite (HA) [13], [14], glass ceramics [10], [15], and composite materials [16], [17] have been intensively studied due to their compatibility with living bone through interfacial formation of a HA interface layer [18]. Taking the biocompatibility and biodegradability into account, bioceramics have been chosen as a promising candidate for potential bone tissue engineering applications. Silicate bioceramics have received significant attention in the past several years due to the fact that they can efficiently stimulate the proliferation, differentiation, and osteogenic gene expression of tissue cells as well as the regeneration of bone tissue by release of Si-containing ionic products [19]–[21] and their special surface composition renders them the ability to be used as a template for the formation of artificial bone tissue [22], [23].

Laponite (Na^+^_0.7_(Si_8_Mg_5.5_Li_0.3_)O_20_(OH)_4_]^−^_0.7_, LAP) is a kind of synthetic silica clay material that can be degraded into nontoxic products under physiological conditions [24], [25]. LAP is biocompatible and has been used as a drug carrier because its interlayer space can be used to encapsulate drug molecules with high retention capacity [24]–[27]. In our previous study, we fabricated poly(lactic-co-glycolic acid) (PLGA) nanofibers incorporated with LAP nanodisks for osteogenic differentiation of human mesenchymal stem cells (hMSCs) [28]. We show that the incorporated LAP is beneficial to promote the cell adhesion and proliferation when compared with pure PLGA nanofibers. More strikingly, the doped LAP within the PLGA nanofibers is able to induce the osteoblast differentiation of hMSCs in growth medium without any inducing factors, such as dexamethasone [28], which is likely ascribed to the fact that the ionic Si and Mg can be released from LAP.

In this study, we prepared LAP ceramic by sintering LAP powder compact at 1200°C for 6 h for potential bone tissue engineering applications. The sintering behavior, mechanical properties, and other physical properties including line shrinkage, relative density, and contact angle of LAP bioceramic under different temperatures, heating rates, and soaking time periods were investigated. The surface morphology of the LAP ceramic was observed using scanning electron microscopy (SEM). The hemocompatibility of the LAP ceramic was investigated via hemolysis assay, while the cytocompatibility of the material was evaluated via resazurin reduction assay as well as SEM observation of the morphologies of rat MSCs (rMSCs) cultured onto the LAP bioceramic. The potential bone tissue engineering applicability of LAP bioceramic was explored by studying its surface mineralization behavior via soaking in simulated body fluid (SBF), as well as the osteogenic differentiation of rMSC cultured onto the material. Finally, the *in vivo* bone defect repair ability and biosafety were studied using a pig model. To our knowledge, this is the first report concerning the preparation of LAP bioceramics for bone tissues engineering applications.

## Materials and Methods

### Materials

LAP with a diameter of 50 nm and a thickness of 7 nm was purchased from Zhejiang Institute of Geologic and Mineral Resources Co., Ltd. (Hangzhou, China). Eagle's Minimal Essential Medium (α-MEM), Dulbecco's Modified Eagle's Medium (DMEM), fetal bovine serum (FBS), phosphate buffer saline (PBS), penicillin, and streptomycin were purchased from Gibco (Carlsbad, CA). β-Glycerophosphate (β-GP), ascorbic acid, resazurin, p-nitrophenyl phosphate, and p-nitrophenol standard were from Sigma (St. Louis, MO). Reporter Lysis Buffer and Picogreen DNA quantification kit were from Molecular Probes, Inc. (Eugene, OR). rMSC and heparin-stabilized pig blood was kindly provided by Shanghai First People's Hospital (Shanghai, China). All other chemicals were from Sinopharm Chemical Reagent Co., Ltd (Shanghai, China) and used as received. Water used in all experiments was purified using a Milli-Q Plus 185 water purification system (Millipore, Bedford, MA) with resistivity higher than 18 MΩ·cm.

### Preparation of LAP bioceramic

LAP bioceramic was produced by uniaxial pressing of 0.45 g LAP powder which was placed in a mold with a diameter of 14 mm under 10 MPa and sintering in a roasting furnace (P300, Nabertherm, German) at different temperatures, heating rates, and sintering time periods (Table 1). Finally, the formed LAP bioceramic was cooled down to room temperature and stored in a desiccator before use.

**Table 1:** Physicochemical parameters of LAP compact and LAP-bioceramic (all data are given as mean ± SD, n = 3).

Sample ID	1*	2	3	4	5
Sintering temperature (°C)	-	600	800	800	800
Heating rate (°C/h)	-	5	5	10	5
Sintering time (h)	-	6	2	6	6
Line shrinkage (%)	-	3.62±0.37	5.99±0.65	7.94±0.65	8.80±1.34
Relative density (%)	-	72.7±1.8	86.5±2.2	87.7±3.1	96.2±2.4
Contact angle (°)	-	27.62±2.33	29.64±0.96	25.29±3.22	18.37±1.24

### Characterization

The surface morphology of various LAP bioceramic was observed using SEM (JEOL JSM-5600LV, Japan) with an accelerating voltage of 10 kV. All samples were sputter coated with gold films with a thickness of 10 nm before observation. The diameter of each sample before and after sintering was measured using a micrometer, and the line shrinkage was calculated by dividing the diameter difference before and after sintering by the diameter of the original LAP compacts. The relative density of each sample after sintering was determined by dividing the apparent density measured in water using the Archimedean technique by the density of LAP powder (2.60 g/cm^3^) [29]. The surface hydrophilicity of LAP bioceramic was evaluated via water contact angle test using a contact angle goniometer (DSA-30, Kruss, Germany). Before analysis, 1 µL water was dropped onto the surface of each sample at the randomly selected areas in triplicate. The contact angle was recorded when the droplet was stable at ambient temperature and humidity. The mechanical property of LAP bioceramic was studied via nanoindentation experiments using a nano indenter (Agilent, Nano Indenter G200, Santa Clara, CA). A diamond Berkovich indenter with a tip radius of 20 nm was used. The constant value of Poisson ratio was 0.25, the vibration frequency of indenter was 45 Hz, and the maximum indentation depth was 4000 nm. The sintering behavior LAP compacts under different temperatures, heating rates, and soaking time periods were comparatively investigated by X-ray diffraction (XRD) using a Rigaku D/max-2550 PC XRD system (Rigaku Co., Tokyo, Japan) using Cu Kα radiation with a wavelength of 1.54 Å at 40 kV and 200 mA.

### Hemolysis assay

The hemocompatibility of the formed LAP bioceramic was examined via hemolysis assay according to our previous study [28]. Briefly, pig red blood cells (pRBCs) were obtained by removing the serum via centrifugation (5000 rpm, 3 min) and washing with PBS for 3 times. The obtained pRBCs were 10 times diluted with PBS. Each sample was placed in the individual well of a 24-well tissue culture plate, and 2 mL of the diluted pRBCs suspension was added. Another two wells containing 0.4 mL of the diluted pRBCs and 1.6 mL of water and PBS solution were set as positive and negative control, respectively. The plate was then incubated at 37°C for 2 h, and the supernatant was centrifuged (10000 rpm, 1 min) and the absorbance of the supernatant related to hemoglobin was recorded using a Lambda 25 UV-Vis spectrophotometer (Perkin Elmer, Waltham, MA) at 541 nm. The hemolytic percentage (HP) can be calculated using the following equation [30],
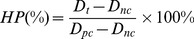
(1)where D_t_ is the absorbance of the test samples; D_pc_ and D_nc_ are the absorbances of the positive and negative controls, respectively.

### Biomineralization

The formed LAP bioceramic (Sample #5, Table 1) was immersed into a 1.5-times concentrated simulated body fluid (SBF) at 37°C up to 7 days, and the SBF solution was changed every 24 h [31]. The LAP bioceramic was removed from the SBF solution after 7 day incubation, gently rinsed with water, and air-dried at room temperature. The formation of HA onto the LAP surface was confirmed using SEM and energy-dispersive spectroscopy (EDS, IE300X, Oxford, U.K.) attached to the SEM equipment.

### Serum adsorption onto LAP bioceramic

The serum adsorption onto the surface of LAP ceramic was quantified according to procedures described in our previous study [28]. Briefly, LAP bioceramic exposed by UV light for 2 h was fixed in a 24-well tissue culture plate (TCP). After that, 1 mL FBS (10%, in PBS) solution was added to each well and incubated for 24 h at 37°C. TCP without LAP bioceramic was set as control. The concentration of FBS before and after adsorption was quantified using a Lambda 25 UV-Vis spectrophotometer at 280 nm based on the FBS calibration curve at the same wavelength. The adsorbed FBS on the surface of LAP bioceramic was also observed by SEM with an accelerating voltage of 10 kV.

### rMSC culture and seeding

rMSCs (passage 2) were cultured in 25 cm^2^ tissue culture flasks with 5 mL complete medium (*α*-MEM supplemented with 10% FBS, 1% ascorbic acid solution (5 mg/mL in PBS), and 1% β-GP solution (1 M in PBS), 100 U/mL penicillin, and 100 U/mL streptomycin) in a humidified incubator with 5% CO_2_ at 37°C. The culture medium was replaced every 3 days and cells were 1∶3 passaged when reaching a confluence of 80–90%. Before cell seeding, LAP was sterilized after exposure under UV light for 2 h. TCPs were set as control. rMSCs (passage 3) were seeded at a density of 2×10^4^ cells per well with 1 mL α-MEM per well and incubated at 37°C and 5% CO_2_. The medium was replaced every 3 days.

### Metabolic activity of rMSCs

The metabolic activity of rMSCs cultured onto LAP bioceramic was evaluated using resazurin reduction assay. At each predetermined time point, medium was replaced with 900 µL complete *α*-MEM and 100 µL resazurin solution (1 mg/mL in PBS). Then the plate was incubated for another 4 h, and the fluorescence intensity in proportion to the viability of the cells was measured by a BioTek Synergy 2 multilabel plate reader (λ_ex_ = 530 nm, λ_em_ = 590 nm).

The morphology rMSCs cultured onto the LAP bioceramic surface after 14 days was observed by SEM with an accelerating voltage of 10 kV. Before observation, cell samples were rinsed 3 times with PBS solution to remove non-adherent cells, and then fixed with 2.5 wt% glutaraldehyde at 4°C for 2 h, followed by dehydrating through a series of gradient ethanol solutions of 30%, 50%, 70%, 80%, 90%, 95%, and 100%. After air dried overnight, samples were sputter coated with a 10 nm thick gold film before SEM observation.

### Alkaline phosphatase activity and DNA content assay

After 14 day culture, rMSCs cultured in 24-well plate were rinsed with PBS for 3 times. After that, 200 µL Reporter Lysis Buffer was added to each well to lyse cells according to the manufacturer's instruction. The cell lysates were stored at −20°C before analysis. For the alkaline phosphatase (ALP) activity assay, 20 µL of the cell lysates was mixed with 200 µL of ALP substrate and incubated for 1 h at 37°C in the dark. Thereafter, 10 µL of 0.02 M NaOH was added to each well to stop the hydrolysis. For comparison, 220 µL of ALP substrate mixed with 10 µL of 0.02 M NaOH in triplicate was used as a blank control. The absorbance was read at 405 nm and the ALP content was calculated from a standard calibration curve.

The DNA content of each cell sample was quantified using Picogreen DNA kit. Briefly, 20 µL cell lysates was mixed with 80 µL Tris-EDTA buffer and transferred to a clean 96-well plate. Then, 100 µL Picogreen working reagent was added to each well and incubated at room temperature for 5 min. The fluorescence intensity was immediately monitored by a BioTek Synergy 2 multilabel plate reader (λ_ex_ = 485 nm, λ_em_ = 538 nm). The DNA content was calculated from a standard calibration curve.

### Alizarin red staining

Before histochemical assay, cells were first fixed with 3.7% formaldehyde in PBS solution for 2 h at 4°C and then rinsed with water for 3 times to remove all traces of formaldehyde. The fixed cells were first covered with Alizarin red S solution (1%, in water with a pH range of 6.3–6.4 adjusted using 0.28% NH_4_OH) for 2 min, washed with water and acidic ethanol (1 part of concentrated HCl to 10000 parts of ethanol 95%), and then observed using Leica DM IL LED inverted phase contrast microscope with a magnification of 200× for each sample.

### *In vivo* biosafety evaluation

All animal studies (including the acquisition of rMSCs and heparin-stabilized pig blood as mentioned in “Materials” section) were approved by the Animal Ethics Committee of Shanghai Jiao Tong University School of Medicine (project number 2012008) according to Regulations for the Administration of Affairs Concerning Experimental Animals (approved by the State Council of the People's Republic of China) and Guide for the Care and Use of Laboratory Animals (Department of Laboratory Science, Shanghai Jiao Tong University School of Medicine, laboratory animal usage license number SYXK 2008-0050, certificated by Shanghai Committee of Science and Technology). Female SD rats (180–205 g) were obtained from Shanghai SLAC Laboratory Animal Co., Ltd. (Shanghai, China). LAP bioceramic was soaked in saline in a concentration of 2 g/mL under 37°C for 3 days, and the biomaterial extract was then sterilized through a filter (Millipore, 0.22 µm) and stored at 4°C. *In vivo* biosafety of LAP bioceramic was evaluated using acute systemic toxicity test and intramuscular stimulation test, respectively. For the acute systemic toxicity test, six female SD rats (divided into two groups, three for experimental group and three for control group) were chosen for the acute systemic toxicity test. Rats in experimental group were intraperitoneally injected with the prepared extract with a dose of 50 mL/Kg according to body weight, while the control group was treated with saline in the same manner. The general toxic effects including appetite, breathe, movement, body temperature, body weight, and survival rate were monitored daily during the first week. For the intramuscular stimulation test, another female SD rat was used and the hair on the back was removed. Then 12 dots on the back were injected with 100 µL saline (dots 1–6, negative controls), 100 µL alcohol (dots 10–12, positive controls, 5% v/v), and 100 µL extract (dot 7–9), respectively. Erythema, edema, and necrosis of skin around the injection region were monitored immediately and at 1, 2, and 3 days post injection.

Histological analyses were performed to further evaluate *in vivo* biosafety of LAP bioceramic extract. Briefly, female SD rat treated with saline or LAP bioceramic extract were euthanized after 14 days, major organs including the heart, liver, spleen, lung, and kidney were harvested and fixed with 10% neutral buffered formalin. Then, the above organs were embedded in paraffin, sectioned into slices with a thickness of 8 µm, and stained with hematoxylin and eosin (H&E). Each stained slide was examined using a Leica DM IL LED inverted phase contrast microscope with a magnification of 100×.

### Animal experiments

Two mature male pigs (Bama miniature swine, 25 kg, 10 months old) were used. Each pig was anesthetized using a mixture of ketamine hydrochloride (60 mg/kg body weight) and xylazine (6 mg/kg body weight). When the animals were in supine position, a rectangular bone defect with a dimension of 1.0 cm×0.2 cm×0.5 cm (length×width×depth) was prepared on the right (control group) and left front (experimental group) leg diaphysis using a orthopaedic bone drills, respectively. Note that this is a non-weight bearing model of assessment. The defect of the experimental groups was implanted with LAP bioceramic while the control group was not implanted with any additional materials. After implantation, penicillin was injected intramuscularly to avoid wound infections. Radiographs of the implant region for each animal immediately after surgery and 24 weeks after euthanized were obtained by X-ray (GE OEC 9900 Elite) under standardized conditions: 60 kV, 4 mA, 4.5 seconds exposure time, 65 cm film-radiation beam distance. Each radiograph was calibrated at the same grey scale and the radiographs were converted to digitalized images using a digital camera.

### Statistical analysis

One way ANOVA statistical analysis was carried out to assess the significance of the experimental data. 0.05 was selected as the significance level, the results were indicated with (*) for p<0.05, (**) for p<0.01, and (***) for p<0.001, respectively.

## Results and Discussion

### Preparation and characterization of LAP bioceramic

In our previous study, we have shown that LAP-doped PLGA nanofibers are able to induce the osteoblast differentiation of hMSCs in growth medium without any inducing factors [28], which may be due to the released Si ions from LAP. Inspired by this, we hypothesize that a scaffold produced from LAP powder without any organic components may also induce the osteoblast differentiation. To prove our hypothesis, in this present study, we prepared LAP bioceramic under a high temperature sintering process, which is expected not to compromise the biocompatibility of LAP [32]. The *in vitro* and *in vivo* performances of the formed LAP bioceramic to act as an artificial scaffold for bone tissue engineering were evaluated.

As shown in Table 1, heating rate, sintering time, and sintering temperature are main factors that may have immediate influence on the formed LAP bioceramic. We first comparatively studied the synergistic relationship between these factors and the properties of the formed LAP bioceramic. Apparently, compared to LAP compact (Sample #1), the chosen sintering temperature (not lower than 600°C) can sufficiently render the LAP compact with a ceramic prototype. With the increase of the temperature (Sample #5 versus Sample #2), and sintering time (Sample #5 versus Sample #4), the line shrinkage (p<0.01) and relative density (p<0.05) significantly increased, implying a densification process of the LAP compacts. The densification may lead to a smooth surface, thus a decreased water contact angle. With the increase of heating rate, the densification of LAP compacts may be slowed down, and the line shrinkage and relative density decrease, while water contact angle increases (p<0.01, Sample #5 versus Sample #2). This shows a contrary variation tendency with sintering temperature and time.

**Figure 1:**
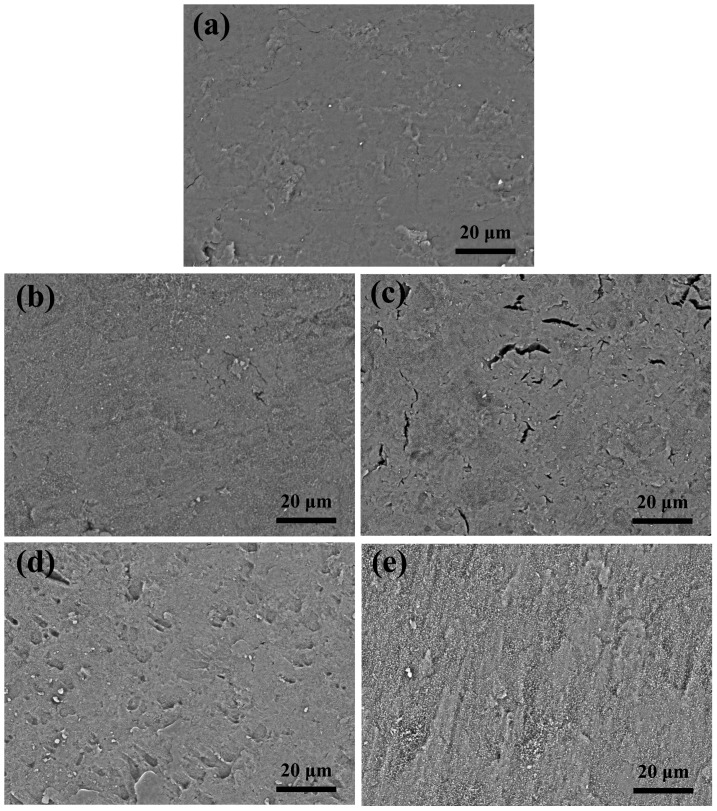
The SEM surface morphologies of the LAP compact. The LAP compact (a) before and (b–e) after sintered. (b) Sample #2, (c) Sample #3, (d) Sample #5, and (e) Sample #4. See Table 1 for sample information.

**Figure 2:**
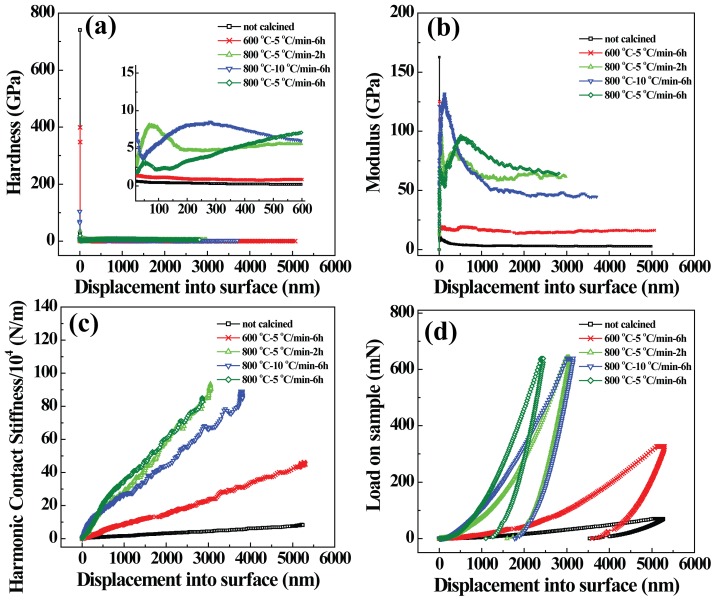
Mechanical properties of the LAP bioceramics. (a) Hardness–displacement, (b) elastic modulus–displacement, (c) harmonic contact stiffness-displacement, and (d) load-displacement curves of LAP compact before and after sintered under different conditions. Inset in (a) shows the hardness change in the range of 20–600 nm.

Figure 1 shows the morphology of LAP compacts and bioceramics under different sintering conditions. Although the LAP compact shows a regular smooth surface, the poor water stability limits its applications. The sintering process does not seem to alter the relatively smooth surface when compared to the LAP compact before sintering (Figures 1b, 1d, and 1e), except the case shown in Figure 1c, where cracks are observed on the surface of LAP bioceramic. It appears that in this case, while the sintering temperature is sufficient to convert the LAP compact to ceramic prototype, however, the sintering time is too short to lead to a smooth surfaced biocermaic. It is notable that some regular small holes can be detected in Figure 1d. With such a rough surface, the attachment and proliferation of stem cells may be facilitated. We then compared the mechanical property of the LAP bioceramic via an instrumented nanoindentation test (Figure 2). The hardness-displacement (Figure 2a) and modulus-displacement (Figure 2b) curves of LAP compact and LAP bioceramics show that both hardness and modulus have an apparent dip in the depth range of less than 20 nm, then increase gradually and level off. Apparently, the final hardness and modulus increase with sintering temperature and sintering time. Figures 2c and 2d show the harmonic contact stiffness-displacement and load-displacement curves. The nearly identical relationship between maximum harmonic contact stiffness and maximum load as a function of sintering temperature and sintering time can be found. Therefore, a optimized hardness, modulus, stiffness, and load can be obtained at a sintering temperature of 800°C, sintering time of 6 h, and heating rate of 5°C/min. LAP ceramics prepared under the optimized conditions was selected for subsequent studies.

**Figure 3:**
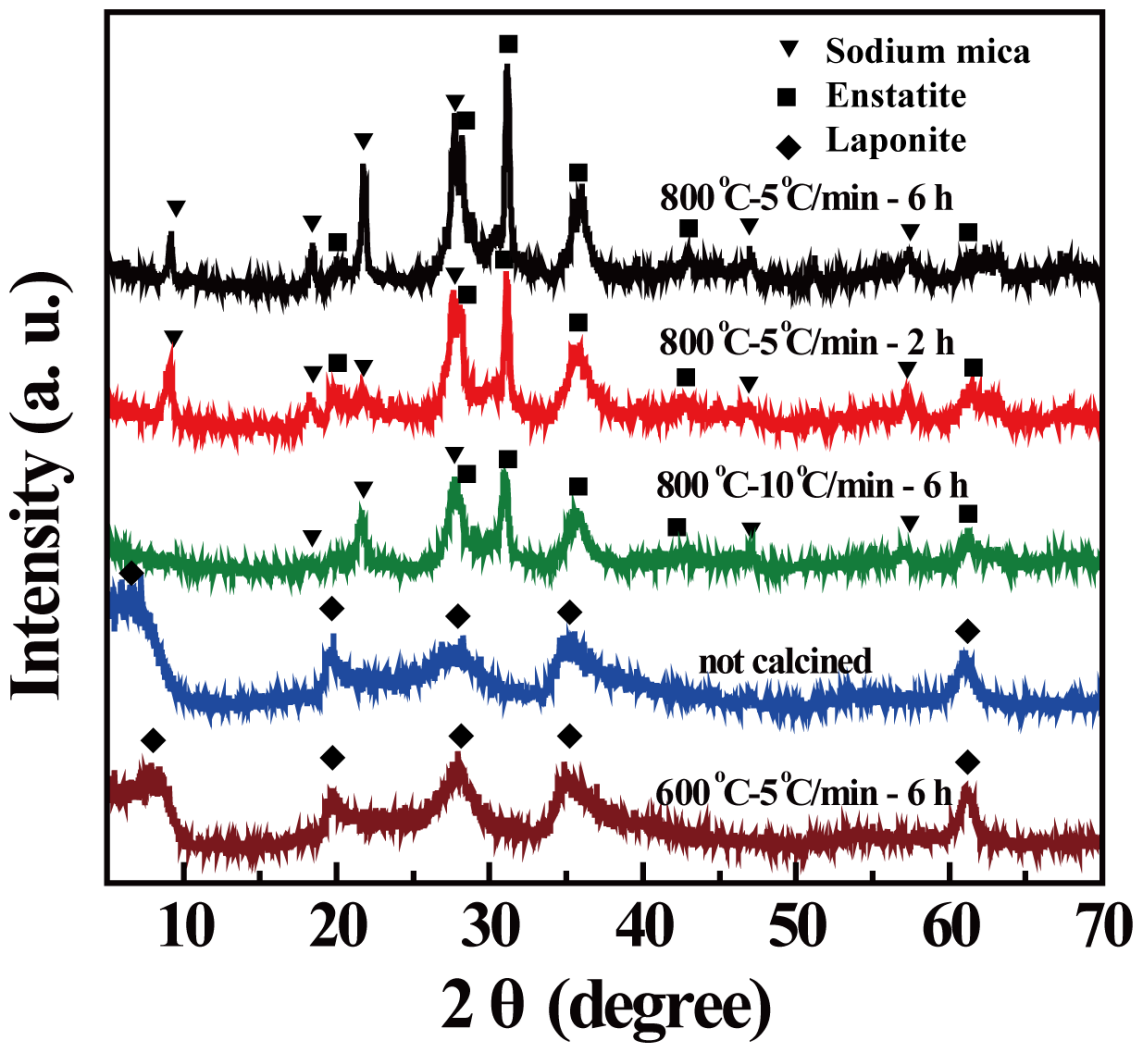
XRD patterns of LAP compact before and after sintering under different conditions.

XRD was used to characterize the crystalline phase change of the LAP compacts before and after sintering (Figure 3). It is obvious that all of the featured LAP peaks exist at a low sintering temperature (600°C). At a high sintering temperature, besides the existing main peaks related to LAP, some new peaks belonging to sodium mica (JCPD: 46-0740) and enstatite (JCPD: 19-0768) emerge in the pattern, illustrating that part of the LAP has been changed into new crystalline phases under a high temperature. Our results indicate that the high-temperature sintering process enables the generation of LAP bioceramic composed of the crystals of LAP, sodium mica, and enstatite.

### Hemocompatibility assay

**Figure 4:**
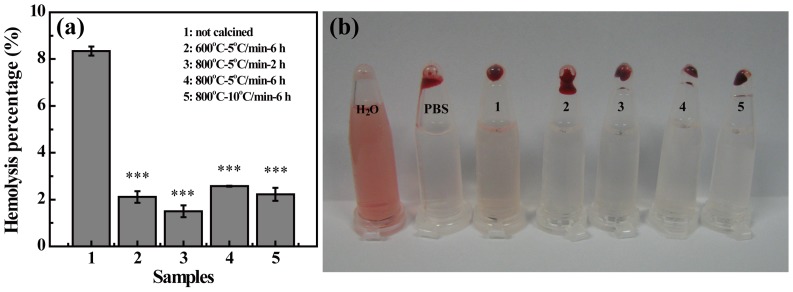
Hemolytic assay data of LAP bioceramics. (a) Hemolytic percentage (%) of pRBCs after treatment with LAP before and after sintered under different conditions for 2 h (mean ± SD, n = 3). (b) shows the photograph of rRBC suspensions after treatment with different LAPs (shown in (a)), followed by centrifugation.

Hemocompatibility has been considered as one of the key issues for an ideal material to be used in tissue engineering applications, especially when the designed scaffold materials are required to contact blood [30]. Like our previous study [28], we evaluated the hemocompatibility of LAP bioceramic via hemolysis assay *in vitro* (Figure 4). Obviously, the pRBCs were totally damaged after exposed to water (Figure 4b, a positive control). In contrast, similar to the pRBCs exposed to PBS solution utilized as a negative control, no visible hemolysis phenomenon was observed after exposure of pRBCs to PBS solution containing LAP bioceramics (p<0.01, Sample #2, #3, #4, and #5, versus sample #1, respectively, as shown in Figure 4a). The hemolytic effects of each sample were quantified by recording the absorbance of the supernatant at 541 nm, which is in proportion to the hemoglobin concentration (Figure 4a). It is found that LAP bioceramics formed under different sintering temperatures have hemolysis percentages all lower than 5% (2.1±0.3%, 1.5±0.3%, 2.6±0.1%, and 2.3±0.3% for Sample #2, #3, #4, and #5, respectively). This indicates that LAP bioceramic possesses good hemocompatibility [33], which is essential for their applications in bone tissue engineering. It is worth noting that the LAP compact without sintering shows a slight hemolysis effect (hemolysis percentage of 8.3±0.2%, Figure 4a; pRBCs was partially destroyed, Figure 4b, tube 1), which may be due to the fact that the degradation products destroyed part of the pRBCs. Our results suggest that a sintering process enables the LAP with improved hemocompatibility, and degradation products of in vivo inserted LAP implant at a relatively high concentration may generate certain degree of hemolysis.

### HA formation and serum adsorption onto LAP bioceramic

**Figure 5:**
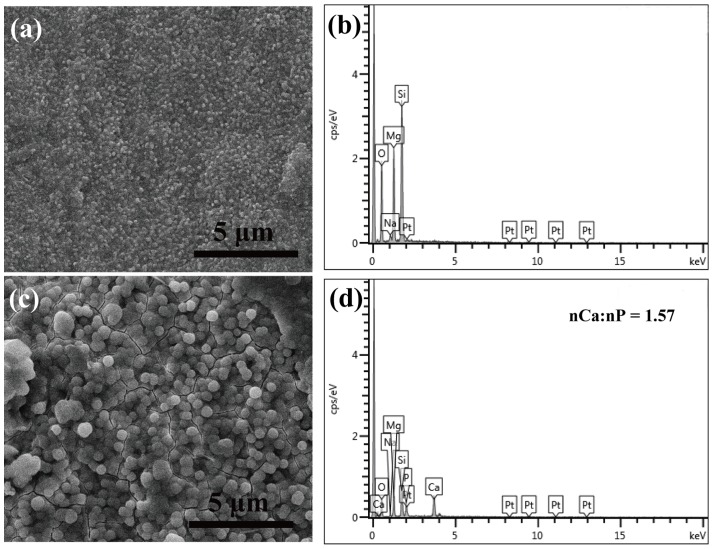
Biomineralization onto the surface of LAP ceramic. (a) and (c) show the SEM surface morphology of LAP ceramic before and after soaking in SBF solution for 7 days, respectively. (b) and (d) show the EDS analysis of the LAP ceramic before and after soaking in SBF solution for 7 days, respectively.

Bone-like HA plays an essential role in the formation, growth, and maintenance of the tissue-biomaterial interface [34]. Bioceramic was reported to be compatible with living bone through interfacial formation of HA interface layer [18]. We then evaluated the HA formation ability by soaking LAP bioceramic into SBF. Figures 5a and 5c illustrate the surface morphology of LAP ceramic before and after soaking in SBF solution for 7 days, respectively. Obviously, the HA deposits could be found on the LAP bioceremic surface after 7 day incubation in SBF. The mineralized HA shows a particulate morphology, with a diameter of nearly 1 µm. Figures 5b and 5d show the EDS analysis of the LAP ceramic before and after soaking in SBF solution for 7 days, respectively. It can be seen that before soaking in SBF, the element of Si, Mg, Na, and O belonging to the LAP itself can be detected. After mineralization for 7 days in SBF, besides the above elements associated with LAP, Ca and P with a molar ratio of 1.57 similar to the stoichiometric molar ratio of HA can be found, further confirming that LAP biocermic possesses the apatite formation ability, which is essential for its bone tissue engineering applications.

**Figure 6:**
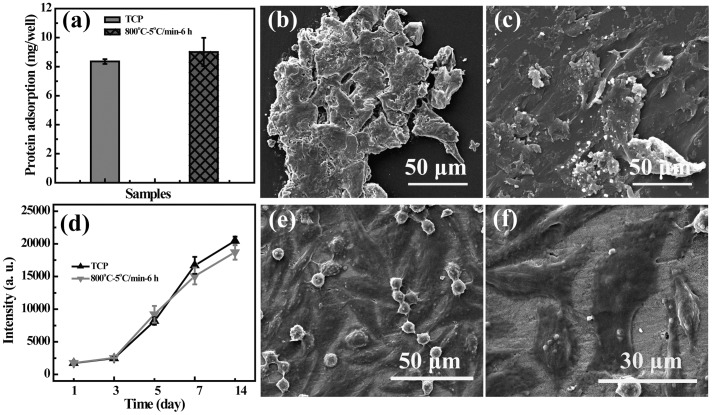
Protein adsorption and metabolic activity assay of rMSCs. (a) The adsorption of protein onto TCP and LAP ceramic (mean ± S.D., n = 3). (b) and (c) show the SEM micrographs of the TCP and LAP ceramic with protein adsorption, respectively. (d) shows the metabolic activity assay of rMSCs cultured onto TCP and LAP ceramic (mean ± S.D., n = 3). (e) shows the micrograph of rMSCs proliferated onto the LAP bioceramic for 14 days. (f) is the magnified image of (e).

An ideal material for bone tissue engineering applications should also have the ability to absorb protein onto its surface, thus providing enough nutrition for cell growth and migration [35]. Based on this point, we then explored the serum (FBS) adsorption capacity of LAP bioceramic (Figures 6a–c). As shown in Figure 6a, the LAP bioceramic can absorb similar amount of FBS when compared to TCP after 24 h incubation (9.1±0.9 mg/well versus 8.4±0.2 mg/well, p>0.05). The adsorbed FBS was further confirmed by SEM (Figures 6b–c), where solid-state FBS is attached onto the surfaces of both TCP and LAP bioceramic. Since TCP has been already coated with other materials to enhance cell attachment and proliferation, it is reasonable to conclude that the protein adsorption onto LAP bioceramic surface may induce enhanced cellular response, similar to our previous reports [28], [36].

### Cytocompatibility assay of LAP bioceramic

With the good hemocompatibility and excellent protein adsorption capacity, we next explored the potential to use the LAP bioceramic as a scaffold for proliferation and osteogenic differentiation of rMSCs, which is important in bone tissue engineering applications. We first analyzed the metabolic activity of the rMSCs cultured onto LAP bioceramic. Figure 6d shows the resazurin reduction assay data of rMSCs at different time points. Apparently, during the first 3 days, the rMSCs grow slowly. During day 3 to day 14, rMSCs experience a typical exponential phase growth [37] and the metabolic activity is enhanced rapidly likely due to autocrine secretion of extracellular matrix (ECM) from cells. Importantly, metabolic activity of rMSCs cultured onto both TCP and LAP bioceramic does not show any significant difference at each time point (p>0.05), implying the good cytocompatibility of the prepared LAP bioceramic.

The morphology of rMSCs seeded onto LAP bioceramic after 14 day culture was observed by SEM (Figures 6e–f). Clearly, rMSCs could adhere onto the LAP bioceramic tightly, confirming that the porous surface morphology is favorable for cell adhesion and proliferation, in agreement with the metabolic activity assay results. A higher magnification SEM image clearly reveals the cell pseudopodia structure (Figure 6f). The rMSCs grown onto the porous LAP bioceramic have filopodia extended and migrated onto the surface to form structured 3D cell-scaffold network, suggesting that the bioactive LAP bioceramic can promote the cell attachment and proliferation. Overall, the sintered LAP bioceramic with porous surface structure, good hemocompatibility, excellent protein adsorption capacity, sufficient mechanical durability, and good cytocompatibility may serve as a porous scaffolding material for bone tissue engineering applications.

### *In vivo* biosafety of LAP bioceramic

**Figure 7:**
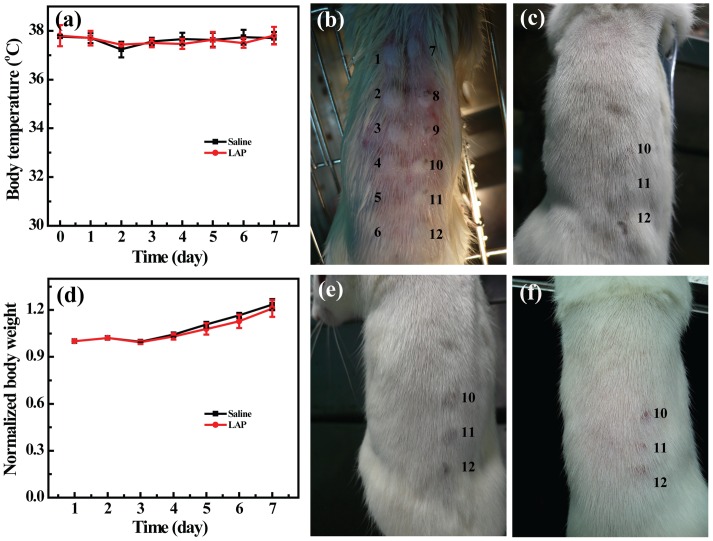
Biosafety test of LAP ceramic extract. (a) Body temperature and normalized body temperature curves of SD rat treated with saline or LAP ceramic extract. (b), (c), (e), and (f) show the results of acute toxicity on normal skin of SD rat immediately after injection or 1 d, 2 d or 3 d post injection of saline (dots1–6), LAP ceramic extracts (dots 7–9), and alcohol (dots 10–12), respectively.

**Figure 8:**
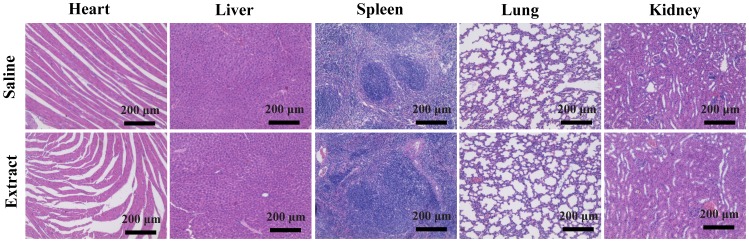
Histological examination. H&E-stained tissue sections of major organs, including the heart, liver, spleen, lung, and kidney from mice treated with saline or LAP ceramic extract for 14 days.

We nest explored the *in vivo* biosafety of LAP bioceramic through the use of acute systemic toxicity test and intramuscular stimulation test, respectively. As shown in Figures 7a and 7d, after intraperitoneal injection of the extract, no obvious body temperature and body weight alteration can be detected between rats treated with saline and LAP bioceramic extract within the first week (p>0.05, rats treated with saline versus rats treated with LAP at each time points). And no death and other toxic symptoms such as appetite reduction, breathing difficulty and mobility impairments were shown for either saline or the extract groups (data not shown). Intramuscular stimulation results (Figures 7b, 7c, 7e, and 7f) illustrate that there is no obvious erythema, edema and necrosis for normal rat skin treated with the extract (dots 7–9, Figures 7b, 7c, 7e, and 7f), similar to that treated with saline (dots 1–6, Figures 7b, 7c, 7e, and 7f) at all monitored time points. In sharp contrast, the rat skin treated with alcohol (dots 10–12, Figures 7b, 7c, 7e, and 7f) displayed obvious skin damage at the injected area. H&E staining was further used to assess the in vivo biosafty of LAP bioceramic, as shown in Figure 8. Compared with rat treated with saline (control group), all of the studied organs from mice treated with LAP bioceramic extract showed no appreciable abnormality or noticeable damage, further suggesting a good in vivo biocompatibility of LAP bioceramic. Our results suggest that LAP bioceramic has no irritation to normal skin of rats, thus possessing an excellent *in vivo* biosafety.

### *In vitro* and *in vivo* bone formation ability of LAP bioceramic

**Figure 9:**
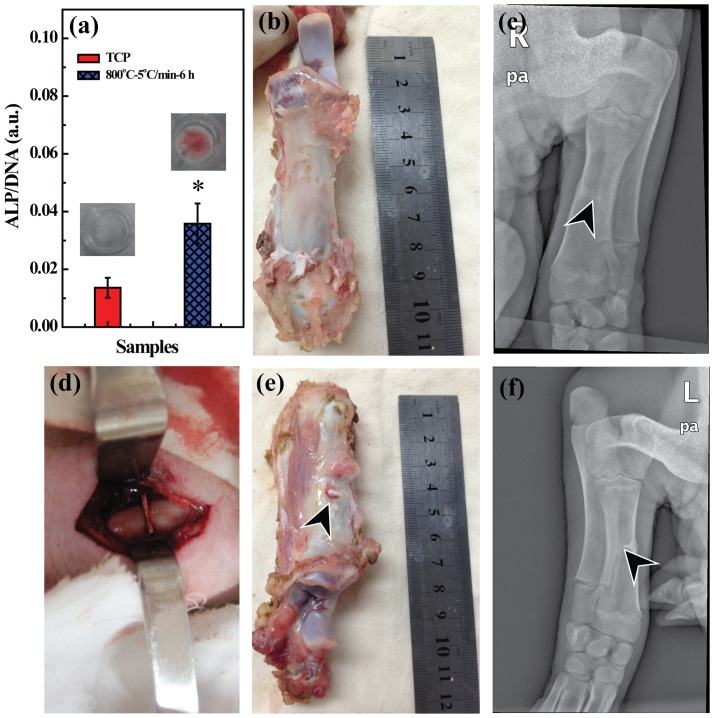
*In vitro* osteogenic differentiation of rMSCs and *in vivo* bone repair evaluation. (a) ALP activity (normalized for the DNA content, nmol of transformed substrate per unit of time and per mass of DNA) of rMSCs cultured onto different substrates in growth medium after 14 days culture. Insert of (a) shows the picture of alizarin red staining of rMSCs cultured onto TCP (left) and LAP ceramic (right) in growth medium without any inducing factors on day 14. (b–f) show the macroscopic appearance of bone defects (A 2-mm bone defect was created in the middle of the tibia, which was implanted with laponite ceramic as shown in (d)). (b) and (e) show the macroscopic appearance of defects without and with implantation for 24 weeks, a trace of laponite ceramic residual is observed in (e) as pointed by an arrow. (c) and (f) show the radiographic images of bone defects without and with LAP implantation after 24 weeks.

We finally explored the ability of LAP bioceramic in regulating the osteogenic differentiation of rMSCs. ALP enzyme is an important marker of osteogenesis which is usually used to monitor the osteogenic differentiation of osteoblasts. It is highly active in osteoblasts involved in the early initiation of mineralization of newly formed bone tissue [38]. We then measured the ALP activity of rMSCs on day 14, and the results (normalized for the total DNA content) are shown in Figure 9a. It is clear that rMSCs cultured onto LAP bioceramic show a significantly higher ALP activity (p<0.05) than those cultured onto TCP in growth medium without any inducing factors on day 14. To further qualitatively confirm the osteoblastic differentiation of rMSCs cultured onto LAP bioceramic, alizarin red staining was performed. Alizarin red S is an ionic dye which tends to bind with the calcium deposition associated with the osteoblastic differentiation and generates a visible red complex. As shown in the inset of Figure 9a, only rMSCs cultured onto LAP bioceramic show an obvious red color, implying the formation of calcium deposit during the osteoblastic differentiation, further confirming that LAP bioceramic can induce osteogenic differentiation of rMSCs in growth medium without any inducing factors.

*In vivo* implantation experiment showed that no apparent wound infection was identified during the healing period or at the time of retrieval and all of the pigs survived after the implantation procedures (data not shown). The bone defects of experimental groups was healed (Figure 9e) while control group was not, illustrating that the defect size is large enough to prove our hypothesis, in agreement with the literature [39]. LAP bioceramic was not distinguishable from surrounding bone and a good union at the LAP bioceramic-implanted interface was observed 24 weeks post-surgery except for a trace of residuals (Figure 9e and Figure S1 in Supporting Information, pointed by an arrow head), implying an excellent ability to use LAP bioceramic to induce the bone formation. Moreover, the defects exhibited some resistance on palpation, further indicating that LAP bioceramic is able to induce bone regeneration [40], [41].

The bone formation was then evaluated using X-ray analysis. Figures 9c and 9f display the representative radiographs of control leg and treated leg 24 weeks after the implantation. Obviously, the residual LAP bioceramic can be clearly detected under the X-ray (Figure 9f). However, the density of the implant region in which the LAP bioceramic was degraded is much higher than that of the control leg (as marked by the arrow head), suggesting the strong new bone tissue deposition capacity of the LAP bioceramic. This may be due to the fact that LAP bioceramic is able to induce the bone formation *in vivo*, in accordance with the *in vitro* bone formation assay data (Figure 9a). Overall, our data demonstrated the excellent implant integration and bone reconstruction ability of LAP bioceramic.

## Conclusion

In summary, we report a facile approach to preparing LAP bioceramic via sintering LAP powder compacts for bone tissue engineering applications. The sintering behavior and mechanical properties of LAP compacts under different temperatures, heating rates, and soaking time periods were comparatively investigated. We show that LAP bioceramic with a smooth surface and relatively regular holey structures can be formed at 800°C for 6 h with a heating rate of 5°C/h under air. The formed LAP bioceramic possesses an excellent surface hydrophilicity and protein adsorption behavior. Besides, the LAP bioceramic is quite cytocompatible and hemocompatible, able to induce the HA deposition onto its surface when soaked in SBF, and enables well proliferation of rMSCs on its surface. Most strikingly, the produced LAP bioceramic is able to induce the osteoblast differentiation of rMSCs in growth medium without any inducing factors. With the good bone healing effect demonstrated by *in vivo* experiments and excellent biosafety, the prepared LAP bioceramic holds a great promise for treating bone defects or other applications in bone tissue engineering.

## Supporting Information

Figure S1 **General observation of the leg diaphysis at 24 weeks after treatment.**
(DOC)
