# Supplementary material for: Preparation of Laponite Bioceramics for Potential Bone Tissue Engineering Applications
Source: PLoS One. 2014 Jun 23;9(6):e99585. doi: 10.1371/journal.pone.0099585 (PMC4067276; doi:10.1371/journal.pone.0099585)
Supplement: Figure S1 — General observation of the leg diaphysis at 24 weeks after treatment. (DOC) [file pone.0099585.s001.doc]

**Supporting Information of**

**Preparation of laponite bioceramics for potential bone tissue engineering applications**

Chuanshun Wang,^a,1^ Shige Wang,^b,1^ Kai Li,^a^ Yaping Ju,^a^ Jipeng Li,^a^ Yongxing Zhang,^a^ Jinhua Li,^c^ Xuanyong Liu,^c^ Xiangyang Shi,^b,d**^ Qinghua Zhao^a*^

^a^ Department of Orthopaedics, Shanghai First People’s Hospital, School of Medicine, Shanghai Jiao Tong University, 100 Haining Road, Shanghai 200080, P. R. China.

^b^ State Key Laboratory for Modification of Chemical Fibers and Polymer Materials, College of Materials Science and Engineering, Donghua University, Shanghai 201620, P. R. China.

^c^ State Key Laboratory of High Performance Ceramics and Superfine Microstructure, Shanghai Institute of Ceramics, Chinese Academy of Sciences, Shanghai 200050, P. R. China.

^d^ College of Chemistry, Chemical Engineering and Biotechnology, Donghua University, Shanghai 201620, P. R. China.

Short title: laponite bioceramics for bone tissue engineering

________________________________________________________

* Correspondence: Qinghua Zhao. Department of Orthopaedics, Shanghai First People’s Hospital, School of Medicine, Shanghai Jiao Tong University, 100 Haining Road, Shanghai 200080, P. R. China. Tel: 86-21-37798566; Fax: 86-21-37798591; Email: [sawboneszhao@163.com](mailto:sawboneszhao@163.com)

**Correspondence: Xiangyang Shi. 2999 North Renmin Road, College of Chemistry, Chemical Engineering and Biotechnology, Donghua University, Shanghai 201620, P. R. China. Tel: 86-21-67792656; Fax: 86-21-67792306-804; Email: xshi@dhu.edu.cn


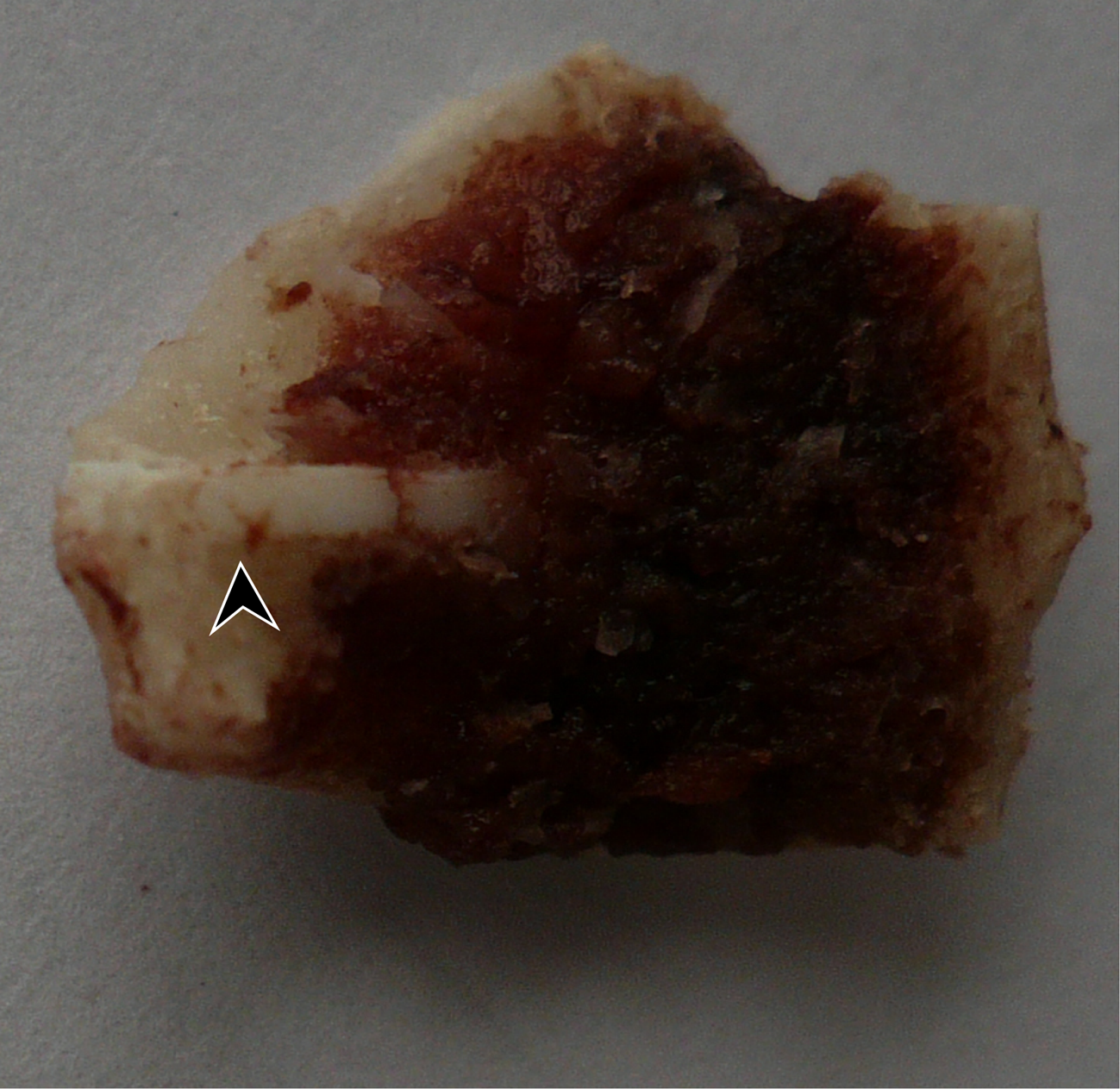


**Figure S1.** General observation of the leg diaphysis at 24 weeks after treatment.
